# A Two-Step Surface Modification Methodology for the Advanced Protection of a Stone Surface

**DOI:** 10.3390/nano14010068

**Published:** 2023-12-26

**Authors:** Liliana Marinescu, Ludmila Motelica, Denisa Ficai, Anton Ficai, Ovidiu Cristian Oprea, Ecaterina Andronescu, Alina-Maria Holban

**Affiliations:** 1 Department of Science and Engineering of Oxide Materials and Nanomaterials, Faculty of Chemical Engineering and Biotechnologies, National University of Science and Technology POLITEHNICA Bucharest, Gh Polizu Street 1-7, 011061 Bucharest, Romania; liliana.marinescu67@gmail.com (L.M.); motelica_ludmila@yahoo.com (L.M.); anton_ficai81@yahoo.com (A.F.); 2 Department of Inorganic Chemistry, Physical Chemistry and Electrochemistry, Faculty of Chemical Engineering and Biotechnologies, National University of Science and Technology POLITEHNICA Bucharest, Gh Polizu Street 1-7, 011061 Bucharest, Romania; ovidiu73@yahoo.com; 3 Academy of Romanian Scientists, Ilfov Street 3, 050054 Bucharest, Romania; 4 Microbiology Immunology Department, Faculty of Biology, University of Bucharest, 1-3 Portocalelor Lane, District 5, 77206 Bucharest, Romania; alina_m_h@yahoo.com

**Keywords:** antimicrobial surface, decoration with silver nanoparticles, stone protection, silanization of natural stone, biofilm inhibition

## Abstract

The biodeterioration of the natural surface on monuments, historical buildings, and even public claddings brings to the attention of researchers and historians the issues of conservation and protection. Natural stones undergo changes in their appearance, being subjected to deterioration due to climatic variations and the destructive action of biological systems interfering with and living on them, leading to ongoing challenges in the protection of the exposed surfaces. Nanotechnology, through silver nanoparticles with strong antimicrobial effects, can provide solutions for protecting natural surfaces using specific coupling agents tailored to each substrate. In this work, surfaces of two common types of natural stone, frequently encountered in landscaping and finishing works, were modified using siloxane coupling agents with thiol groups. Through these agents, silver nanoparticles (AgNPs) were fixed, exhibiting distinct characteristics, and subjected to antimicrobial analysis. This study presents a comparative analysis of the efficiency of coupling agents that can be applied to a natural surface with porous structures, when combined with laboratory-obtained silver nanoparticles, in reducing the formation of microbial biofilms, which are a main trigger for stone biodeterioration.

## 1. Introduction

Silver nanoparticles (AgNPs) find applications across multiple domains, such as medicine, biosensors, biotechnology, the textile industry, wastewater treatment products, construction, detergents and cosmetics, electronics, and antimicrobial packaging [1,2,3,4]. AgNPs are well known for having an inhibitory effect on the most common microorganisms in medical and industrial applications [5,6]. AgNPs are leading nanomaterials in the fight against pathogenic microorganisms, being efficient against planktonic cells and even biofilms [7,8,9]. Unlike the bulk form of silver, AgNPs exhibit a distinct behavior primarily owing to their enlarged specific surface area, these features being responsible for their special behavior in this regard [10,11,12].

Nanomaterials characterized by small sizes and high surface areas possess the capacity to penetrate deep into porous materials, such as stone, and protect their surface against water absorption and the action of bio-deteriorating agents, while improving the strength of the damaged surface [12]. All these properties are essential against the degradation of substrates [11,13,14]. The bactericidal mechanism of action of silver nanoparticles entails multifaceted processes that inflict damage on bacterial cell walls and plasma membranes and also inhibit DNA replication and protein synthesis. This effect is mainly due to silver nanoparticles releasing steadily silver ions onto the surface and favors their biocidal effect at a lower concentration than that in silver salts [14].

Among the diverse applications of silver nanoparticles, the biodeterioration and biodegradation of stone and stone-based materials are important concerns of the scientific community, caused by climatic changes that have a negative effect on buildings, including their claddings, especially when exposed to the outside. In the past years, the preservation of cultural heritage buildings has become an important issue globally due to these more aggressive degradation factors, and thus, it is attracting special attention to find better ways to protect not only cultural heritage buildings but also ordinary buildings [12,13].

Biodeterioration is an undesirable phenomenon caused by microorganisms [15]. Microorganisms, which include bacteria, fungi, algae, and lichens, colonize stone claddings, monuments, etc., as a result of their exposure to the climatic factors of the external environment and cause significant deterioration [16,17]. Biodeterioration can cause chemical, physical, and aesthetic damages as well [18]. Over time, these microorganisms accumulate in biofilms and become more resistant because of their embedding in an organic matrix well adhered to the stone structure. One of the most important features of biofilms is the resistance to antimicrobial agents induced by slow metabolism of certain subpopulations of agents, a biofilm-specific phenotype, large persistent cell populations inside the biofilms that do not die in the presence of microbicidal antibiotics, QS action, and activation under general stress [19]. Thus, the alteration mechanism of stone associated with biodeterioration can be of two types: physical or chemical. The physical alteration mechanisms are those that induce the loss of cohesion of the substrate due to mechanical and chemical alterations. As a consequence, the substrate is altered since microorganisms consume substances/nutrients and excrete products, such as pigments and organic acids [14,20]. Bacteria can live either as free planktonic cells or as sessile cells attached to a surface [21]. Biofilms lead to both surface and in-depth biodeterioration of a substrate. At the same time, fungi play a crucial role in the disintegration of stones due to their enzymatic activity; thus, the colonization of stones with fungi appears spotty or with black crusts [22]. Fungi can synthetize mycotoxins and have the potential to cause biodeterioration, even of inorganic substrates, such as stone [23,24].

The main factors that have major influences on stone surfaces are intrinsic factors (mineralogical and chemical composition, porosity of stones) and extrinsic factors, such as (*i*) atmospheric agents, water humidity in the air, UV rays, sun rays, and acid rains; (*ii*) salt crystallization due to the inadequate installation of a certain stone; (*iii*) biodeterioration caused by the action of various microorganisms; and (*iiii*) anthropic agents, such as vandalism and earthquakes [25]. Microbial colonization on the surface of stone materials leads to a negative impact, including physical and chemical deterioration of the stone. The biodeterioration process is the result of complex activities and interactions of the microbial communities with the stone matrix [14,22,26].

The protection against developing microbial growth on different substrates is performed with a wide range of surface coatings with advantages and disadvantages; thus, a polymeric film confers cohesion between the grains of the stone structure but appears dark and as spots rising from the stone surface [27]. Usually, coating products contain resins that act like adhesive or gluing agents and do not really impregnate or strengthen the stone surface [28]. The application of a coating using various resin materials serves as a protective measure against environmental factors for the stone surface. However, over time, the efficacy of such coatings diminishes due to the fact that they are applied externally, rendering them susceptible to extrinsic factors that exert adverse effects. Furthermore, the imposition of a coating layer onto the stone surface impedes its inherent breathability, thereby hastening the impact of intrinsic deteriorative factors. In general, the resin penetrates much less deeply than silicone resins and displays a chromatic alteration due to UV rays. Removal of biofilms is usually performed using the same techniques, followed by biocidal treatment [29] or impregnation operation with various materials, starting from biocide products to using biofilm formation itself as treatment [30,31]. Endolithic organic matter associated with lichen colonization can provide waterproofing to the stone and act as a sulfate contamination barrier [19].

This research aims to provide valuable insights into the intricate relationships between material properties and the performance of surface treatments for natural stone. The efficiency of silver nanoparticles in the protection of stone substrates by treating two different porous sedimentary stones with siloxane-based coupling agents and silver nanoparticles obtained in the laboratory was evaluated and was proved to ensure long-term protection against biofilm formation [32,33,34,35]. Silver nanoparticles have high antimicrobial activity (bactericidal, fungicidal activity) [12]. Thus, using silver nanoparticles to bind the siloxane coupling agents can act through different mechanisms against microorganism formation and biofilm formation [16]. The use of a mixture comprising a siloxane coupling agent and alcohol, as opposed to using a 100% siloxane coupling agent alone, for fixing silver nanoparticles onto the stone surface reveals that the extent of their adsorption is contingent upon factors, such as the stone type, porosity, and structural characteristics. Both components, nanoparticles and siloxane coupling agents, are used not only for the water repellent capacity but also for the petro-physical properties of the treated stone substrates, the durability of treatment, and color changes, being an assessment for the protection of historical buildings [29,36,37].

## 2. Materials and Methods

### 2.1. Materials

All the nanoparticles used in this work were synthetized in the laboratory based on chemical routes previously published [38,39,40,41]. The selection of colloidal AgNPs was performed based on their antibacterial efficiency. Based on antimicrobial tests [40], the selected colloidal solutions were those obtained via the wet-chemical route at room temperature, coded in this work as sample 1 (s1), and those obtained at room temperature via the solvothermal route, coded as sample 2 (s2). Colloidal AgNP solutions of different concentrations (such as 10 ppm and 1000 ppm) were used. Different types of stone substrates were used for impregnation. The characteristics of the stone substrates are presented in Table 1. The values of water absorption by weight were according to ASTM C1527 for travertine and C 568 for limestone, depending on the provenance of the stones. This characteristic can vary from 2 to 10%, and for young travertine, the values are higher. For limestone, the values of water absorption, depending on the type of grains, are low, medium, or fine. The values can vary between 3 and 12% and usually are less than 10%. Their selection was performed not only based on their use as building materials but also considering their physical and mechanical properties (durability and resistance) [39,40,41,42]. Travertine is mainly formed of calcium carbonate mixed with traces of magnesium oxide, silica, and aluminum oxide with a high water absorption capacity [8,42,43,44]. Limestone is mainly formed of calcium carbonate (CaCO_3,_) and trace amounts of magnesium carbonate (MgCO_3_), silicon dioxide (SiO_2_), iron (III) oxide (Fe_2_O_3_), and magnesium oxide (MgO) [25]. The water absorption characteristics of both investigated stones were ascertained through regression equations emphasizing the establishment of optimal correlation with stone porosity.

Overall, this approach involved coupling agents bearing a proper functional group, ensuring specific molecular interactions that play a critical role in achieving a durable and well-adhered nanoparticle coating on natural stone substrates. The combination of covalent and ionic bonding mechanisms contributes to the effectiveness and longevity of the treatment, making it a promising method for enhancing the properties of natural stone surfaces. One important aspect of the stone treatment is the right selection of coupling agent, which ensures the anchoring of most nanoparticles on the mineral substrate [28,33].

### 2.2. Synthesis of AgNPs

Silver nanoparticles were synthesized using two chemical synthesis methods: the classical method at room temperature and the solvothermal method [38,39,40,41]. According to the antimicrobial results obtained for the two types of AgNPs (Table 2), the solution obtained using the classical reduction method at room temperature at 10 ppm and the solution obtained using the hydrothermal synthesis route at 1000 ppm were used [38].

### 2.3. Stone Surface Modification

The stone samples used were in cubic form with sizes of 1 × 1 × 1 cm^3^ or thin samples with sizes of 1 × 1 × 0.1 cm^3^, depending on the application. The stone samples were first washed with warm water and bicarbonate solution and then with deionized water and dried in the oven at 80 °C for 24 h. Bicarbonate solution was used for washing the stone surface in neutral (pH = 7) medium. The stone samples were cut in cubic shapes of ~1 × 1 × 1 cm^3^ and further treated with coupling agents.

**Figure 1:**
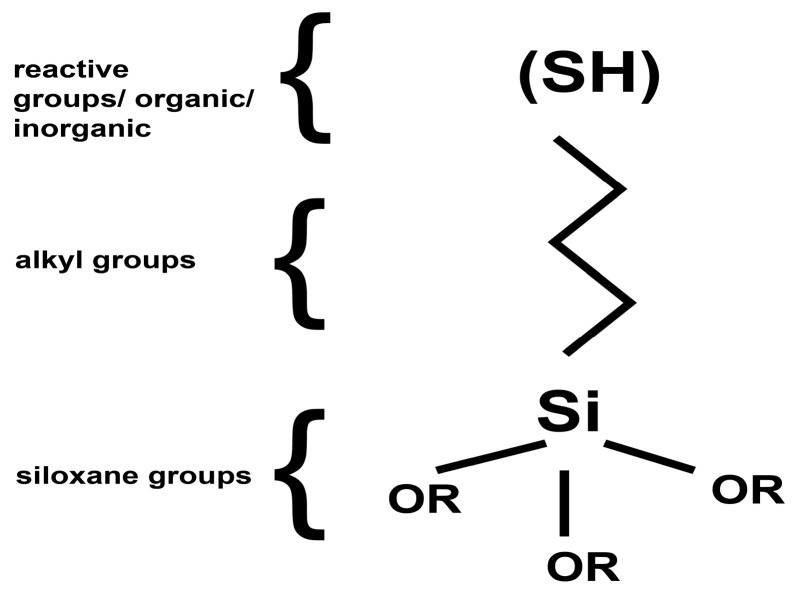
General formulae of silane groups containing an –SH group.

The treatment of natural stone substrates with nanoparticles, particularly using AgNPs, can be carried out by using adequate coupling agents. These coupling agents are intermediary compounds that play a crucial role due to their molecular structure (Figure 1). One end of these compounds attaches to the inorganic surface of the mineral substrate, forming covalent bonds with the hydroxyl (OH) groups originating from the surface. Simultaneously, the other end of these intermediary compounds is composed of thiol groups. Siloxanes with thiolic moieties were chosen as silanization agents, since it is known that silver nanoparticles have a high affinity to the thiol group [30,31]. These thiol groups attach to the AgNPs via coordinative covalent bonding; thus, the nanoparticles are anchored to the mineral substrate, facilitating and changing its various properties [45,46,47].

**Figure 2:**
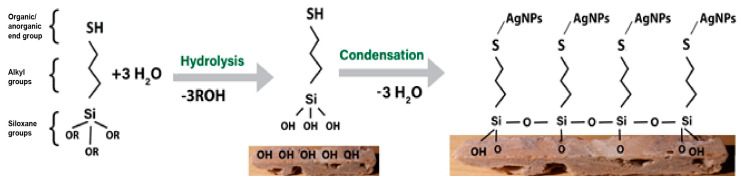
Scheme of stones’ impregnation.

As modification agents, silanes bearing thiol groups were used, namely 3-mercaptopropyl methoxysilane from Aldrich and the commercial product named CoatOSil T-Cure from Momentive, usually used in coatings as an additive (Table 3). The silanization agents possess a hydrolytically sensitive center that can react with inorganic substrates, such as natural stone, according to the reaction presented in Figure 2, leading to stable covalent bonds by which thioalkyl groups are attached to the surface of the stone. The organic groups with –SH end groups (Figure 2) remain on the outer side of the stone surface, conferring new properties, including a high affinity for silver nanoparticles, but without blocking the stone’s pores and let the stone breathe [44,48]. In the first step, all samples were treated with the silanization agent in two variants: (a) pure silanization agent and (b) 50% silanization agent+ 50% i-propanol (IPA). The aim of using i-propanol was to ensure lower viscosity and thus better penetration inside their surface structure (pores and cracks). The impregnation operation was performed using brushing in order to reproduce the everyday working condition.

### 2.4. Characterization of the Obtained Samples

FTIR imaging was performed using a Thermo IN50 MX microscope (Thermo Scientific, Waltham, MA, USA). An FTIR image was used to obtain information both for the stone structure and for the siloxane coupling agent’s presence on the treated surface. The FTIR microscope was operated in reflection mode (germanium ATR unit) to study the samples’ structural features.

The surface morphology of the samples was examined via a QUANTA INSPECT F (FEI- PHILIPS, Eindhoven, The Netherlands) electron microscope equipped with a field emission gun with 1.2 nm resolution and an energy-dispersive X-ray (EDS) detector with 133 eV MnK resolution on samples covered with carbon. SEM was performed on the impregnated stone samples to analyze the surface morphology. The elemental composition was evaluated using EDS.

The UV–Vis spectra were recorded using an Evolution 300 UV–VIS spectrophotometer in absorbance mode (190–1100 nm), selecting a 1 nm data interval, a 2 nm bandwidth, and a 240 nm/min scan speed, in 10 mm quartz cuvettes, at room temperature.

### 2.5. Antimicrobial Evaluation

The antimicrobial activity of AgNPs and siloxanes as coupling agents, deposited on the stone samples, was evaluated against the Gram-positive bacterial strain model *Bacillus subtilis ATCC 6051*. This species was selected because it is a widespread and sporulated bacterium found in soil and the natural environment and can be also an agent for natural stone biodeterioration. *B. subtilis* is a non-fastidious microorganism routinely used for sterility checks and the evaluation of the antimicrobial effect of antimicrobial agents applied on abiotic surfaces [2].

#### Biofilm Inhibition

*B. subtilis* adherence and biofilm inhibition on the nano-treated stone samples was analyzed using a cultivation-dependent method based on viable counts and the determination of colony-forming units (CFU)/mL values. Briefly, 0.5 Mc Farland suspension in sterile saline (1.5 × 108 CFU/mL) was obtained from fresh *B. subtilis* cultured on nutritive agar. Next, 10 mm × 10 mm × 1 mm pieces of various stones (travertine and limestone) treated with the two coupling agents and decorated with AgNPs were sterilized with UV exposure. Sterile stone specimens were aseptically transferred to 24-well plates, and 1.5 mL of nutritive broth was added. Next, 15 μL of *B. subtilis* suspension was added, and the plates were incubated at 37 °C for 24 h. After incubation, the stone specimens were briefly washed with sterile saline solution to remove unattached bacteria, and then, specimens containing *B. subtilis* biofilm cells were transferred to sterile 15 mL Falcon tubes containing 2 mL sterile saline solution. The Falcon tubes were then vigorously vortexed for 20 s and exposed to ultrasound for 10 s to detach the biofilm cells, which were obtained as suspensions. The suspensions containing biofilm cells were then subjected to serial dilutions, and these were inoculated on agar plates to establish CFU/mL values.

## 3. Results and Discussion

The silanization process was performed as an intermediary step in decorating the stone surface with silver nanoparticles, and thus, special attention was paid to the evaluation of the antimicrobial activity induced as a consequence of the decoration of the surface with these nanoparticles.

### Results and Discussion of Impregnated Stones

**Figure 3:**
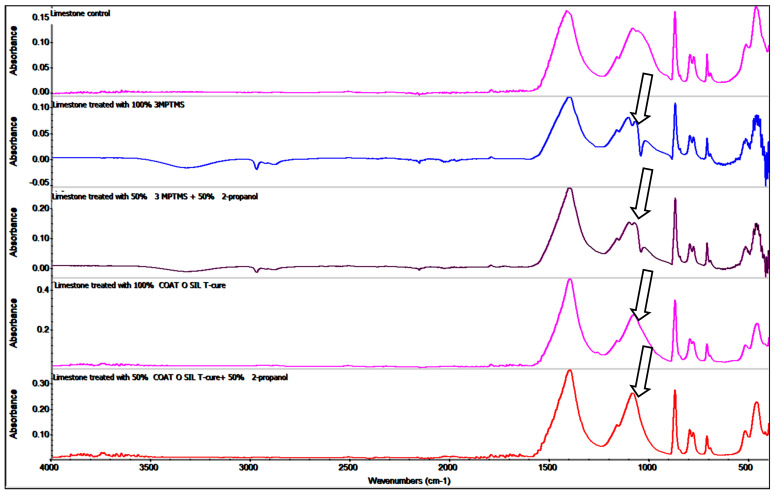
Comparative FTIR images of limestone samples treated with different coupling agents.

**Figure 4:**
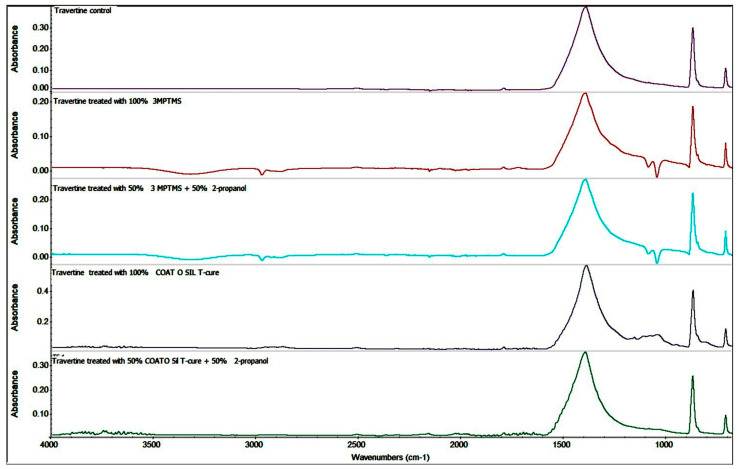
Comparative FTIR spectra of travertine samples treated with different coupling agents.

FTIR images for both variants of stone, named travertine and limestone, treated with the silanization agents were obtained for mineralogical characterization of the powdered samples and compared with samples of untreated stone, named “controls” for both stone samples: limestone (Figure 3) and travertine (Figure 4). FTIR images for all the variants of stones showed characteristic stretching vibrations of calcium carbonate (CaCO_3_) peaks around values of 1409–1500 cm^−1^ and 720–880 cm^−1^ [49,50].

The band located around values of 1440 and 795 cm^−1^ can be attributed to the CO_3_^2−^ anion obtained from calcite. Quartz was a minor constituent and was identified through the band located at 1100 and 790–800 cm^−1^, which is attributed to silicate groups’ vibrations [44]. Peaks located around the value corresponding to 1140–1168 cm^−1^ can be attributed to the presence of silica, one of the components of the limestone structure, and the value of 1140 cm^−1^ can be associated only with silicate groups [49]. Another important component occurring in the structure of limestone, gypsum, calcium sulfate hydrate (CaSO_4_ × 2H_2_O), was indicated by the presence of peaks around values of 1109 and 669 cm^−1^. The silanization with 3-MPTMS and CoatOSil T-Cure was proved by the FTIR spectra modification at about 1140 cm^−1^. The presence of silicate can be seen by overlapping on the quartz band, being nearly placed around a band value of 1040–1000 cm^−1^. The presence of a peak around 1100–1000 cm^−1^ for the limestone control indicates the silica groups being characteristic of the tested limestone (Figure 3). The Si–alkoxy compound had a strong band at 1110–1000 cm^−1^. If siloxanes are present in the sample, the 1110–1000 cm^−1^ band is masked by strong Si–O–Si absorption, as indicated by the arrows (Figure 3). Comparing the FTIR images of the same stone, treated limestone, with different coupling agents, 3-MPTMS and CoatOSil T-Cure, we noticed variation values, indicating the presence of Si–O–Si around values between 1130 and 1050 cm^−1^, and it seems that 3-MPTMS is more reactive, and thus, the intensity of these peaks was stronger on both stone samples [45,51,52].

FTIR spectra recorded for the travertine samples showed the presence of siloxane groups, proving functionalization based on the peaks around 1100–1000 cm^−1^. The strong Si–O–Si antisymmetric stretch mode indicates the presence of values around 900–1300 cm^−1^. The last spectrum of travertine treated with CoatOSil T-Cure/isopropanol highlighted just a shoulder-like absorption in this area, most probably because of better penetration of the silanization agent into the pores, and thus, the concentration of these peaks was lower (Figure 4).

The presence of the peaks around 1110–1000 cm^−1^ corresponding to Si–O–Si and ~970 cm^−1^ corresponding to Si–OH confirm the presence of a coupling agent on the surface of the stones. Being a natural surface with a specific imperfection, with open pores, the impregnation of surfaces showed a variation that confirmed that the quality of treatment is influenced by the type of stone. The IR maps were recorded at different wavelengths (corresponding to CO_3_^2−^ and SiO_4_^4−^). Comparing the maps, the high similarity of these pairs of FTIR images indicated that the modification of the surface of the stones was homogeneously realized.

**Figure 5:**
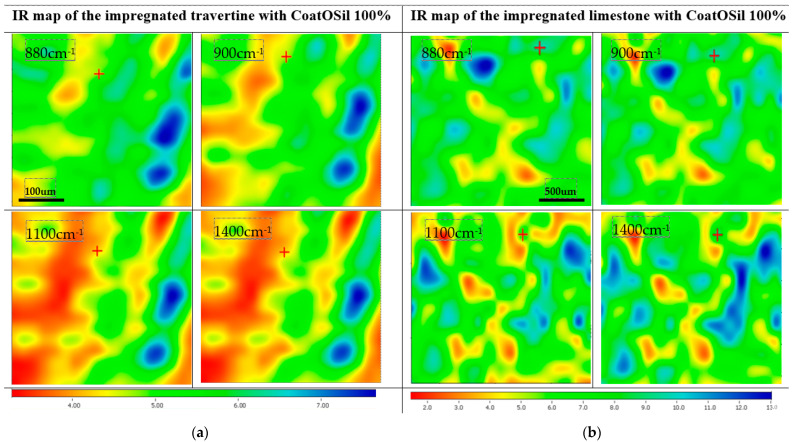
IR maps of travertine (**a**) and limestone (**b**) treated with CoatOSil T-Cure silanization agent.

**Figure 6:**
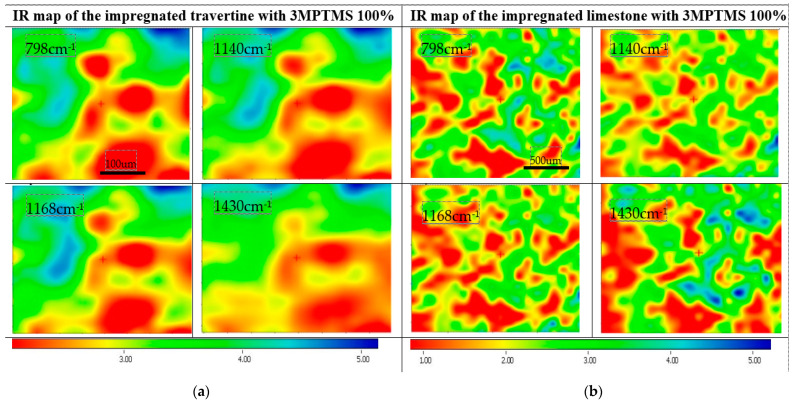
IR maps of travertine (**a**) and limestone (**b**) treated with 3-MPTMS silanization agent.

The surfaces treated with CoatOSil T-Cure, as shown in Figure 5, exhibited a relative uniformity across different wavelengths, demonstrating a subtle distinction between stones types. Notably, the impregnation process implied pure CoatOSil T-Cure appears to introduce greater heterogeneity in the case of travertine, which can be explained based on the higher viscosity and thus lower wettability on the entire surface—including pores. Conversely, when using 3-MPTMS (Figure 6), better homogeneity was obtained with the pure silanization agent. This outcome can likely be attributed to the comparatively lower viscosity characteristic of this commercially available silanization agent.

**Figure 7:**
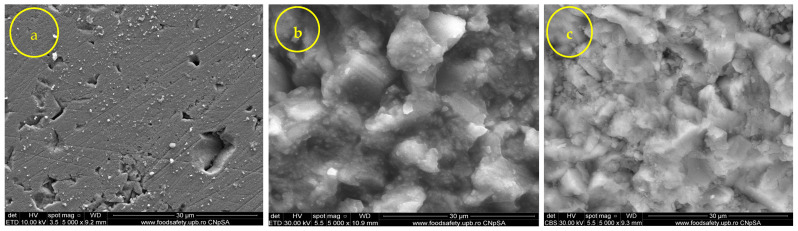
SEM images of different stages of the impregnated stone: (**a**) travertine control, (**b**) travertine treated with CoatOSil T-cure, and (**c**) travertine treated with 3-MPTMS.

**Figure 8:**
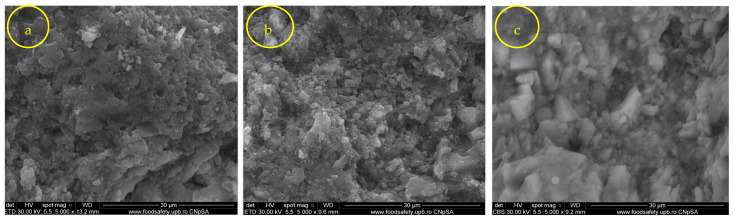
SEM images of different stages of the impregnated stone: (**a**) limestone control, (**b**) limestone treated with CoatOSil T-Cure, and (**c**) limestone treated with 3-MPTMS.

The film morphology was analyzed using SEM analysis. SEM images of untreated and treated samples provided information related to the aspect of surfaces. This technique provides the aspect of the coupling gent in relationship with the treated substrate [27]. The untreated surface (control) in the case of travertine showed a porous surface (Figure 7a). Both coupling agents, 3MPTMS and CoatOSil T-Cure, covered the porous surface of travertine (Figure 7b,c) and gave a smoother aspect than the control. The aspect of the surface treated with the coupling agent is a continuous film-coated surface with no visible pores. Compared with travertine, limestone (Figure 8a) had a less porous surface, and thus, fewer important visual changes (Figure 8b) occurred after the silanization treatment with the two indicated silanization agents (Figure 8b,c). The grains of limestone were of medium size, and the impregnation showed a relative homogeneous penetration of the stone surface, keeping the original aspect of the limestone (Figure 8b,c). The impregnation of the limestone surface with 3-MPTMS showed a film coating similar to the impregnated travertine surface (Figure 8c), with gel formation and attached to both stone surfaces, as noticed for the different impregnation of treated stone [29,52,53,54]. Comparing SEM images obtained for the two types of stones showed that depending on the structure of the stones, the coupling agent should be chosen [14,28]. Both coupling agents showed that they modify the surface, and this can be correlated with the FTIR microscopy, too. The adsorption of the coupling agent depends on the stone structure, and coupling agents thus facilitate the formation of a film as a crust or can penetrate easily the porous surface of certain stones [24]. Based on these observations, it can be concluded that silanization leads to a rough surface, which theoretically can enhance the bacterial colonization, but hopefully, the combination of the coupling agent and silver nanoparticles will lead to a resistant surface against biofilm formation [28].

**Figure 9:**
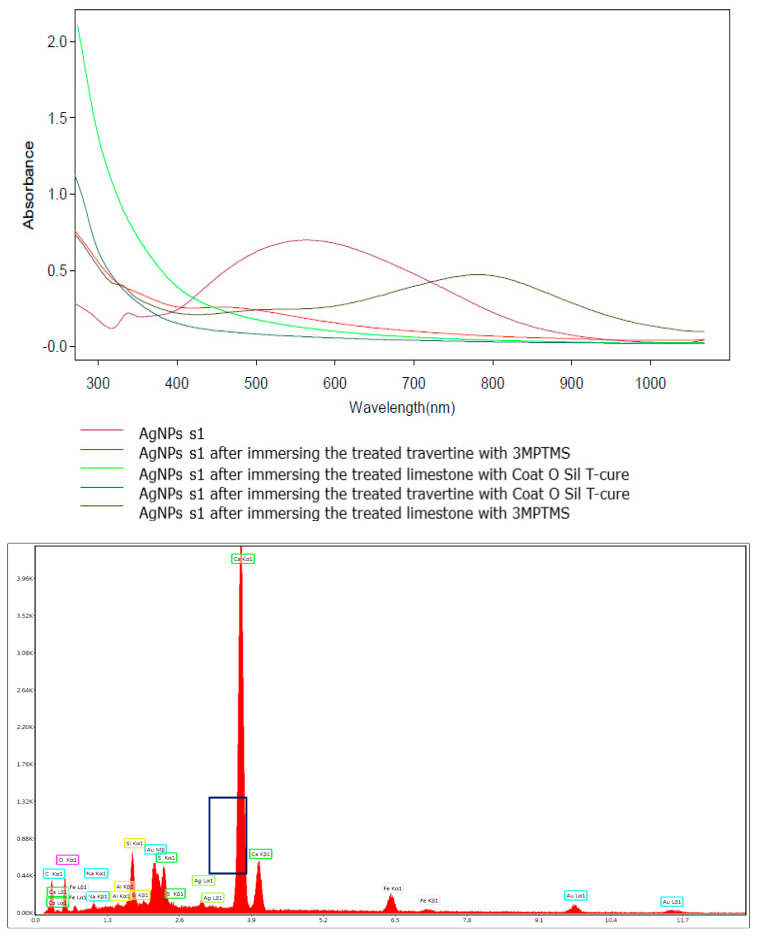
UV–Vis quantitative determination of the presence of AgNP s1 on treated stones and EDS graphic for the limestone treated with CoatOSil T-cure indicates the presence of AgNPs, marked in the black box.

**Figure 10:**
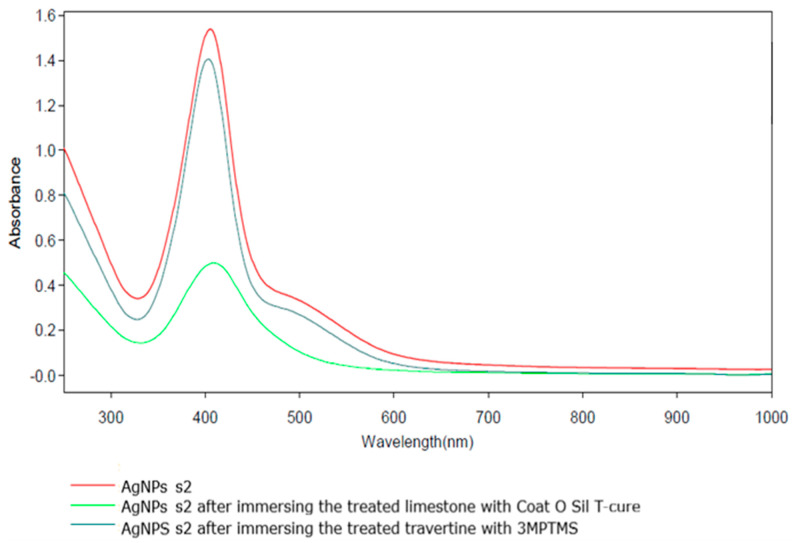
UV–Vis quantitative determination of the presence of AgNP s2 on treated stone.

Because of the low content of nanoparticles, it is important to mention that SEM images cannot reveal the presence of AgNPs [10,54] on samples treated with the above-mentioned silanization agent and further treated with AgNPs at the two concentrations (10 and 1000 ppm). This fact is due to the low concentration of AgNPs, which are also partially adsorbed onto the porous surface. Even though both stones were treated before with the silanization agents, the silver nanoparticles are impossible to be detected using this technique, and this also happens in EDS measurements. Considering the need to quantify the amount of absorbed AgNPs, UV–Vis assessments were considered. The absorption of AgNPs was evaluated considering the initial and the final absorbance of the solutions for each type of AgNPs (s1 and s2) used for the treatment of the stones. Even SEM images cannot prove the presence of AgNPs on the stone surface, and the UV–Vis absorption spectra showed that AgNPs were well adsorbed onto the surface, as revealed from Figure 9 and Figure 10. Considering AgNP s1—the blueish sample with 10 ppm AgNPs (Figure 9)—it is obvious that the absorption of these nanoparticles is strongly dependent on the substrate and functionalization agent. It is worth mentioning that the increased absorption at 300–400 nm after the immersion of the stone samples into the two solutions is a consequence of the ion release from the stones, and thus this part of the spectra was ignored. All the discussions will be conducted only in the visible range, 420–1100 nm. The functionalization with 3-MPTMS for both limestone and travertine did not lead to total absorption of the AgNPs (even if the concentration of the solution was just 10 ppm). In the case of CoatOSil T-Cure, the absorption was almost 100% for the travertine substrate. It was noticed that the AgNP HT with 1000 ppm concentration showed relatively weak absorption on the travertine substrate treated with 3-MPTMS (~8%) but substantial absorption on the limestone substrate treated with CoatOSil T-Cure (60%) (Figure 10).

## 4. Antimicrobial Activity

The antimicrobial assessment was conducted on samples of travertine (T) and limestone (L) after impregnation with AgNPs and the evaluated coupling agents.

**Figure 11:**
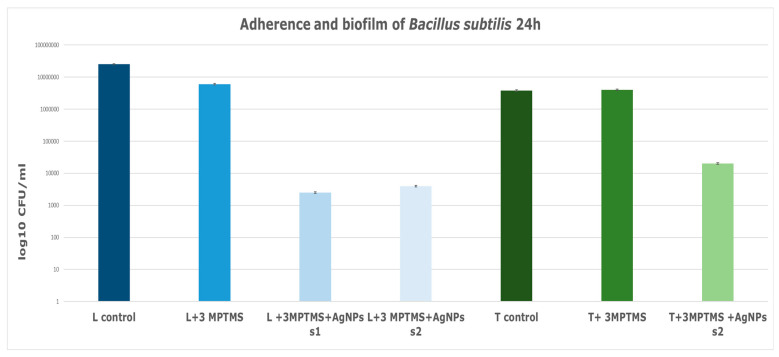
Graphic representation of *B. subtilis* adherence and biofilm inhibition when incubated for 24 h at 37 °C in the presence of stone specimens treated with 3-MPTMS and AgNPs obtained with various chemical methods: s1 obtained via chemical reduction at room temperature and s2 obtained via solvothermal synthesis.

The analysis of *B. subtilis* biofilm adherence on various substrates revealed distinct outcomes. The analysis of both types of stone substrates impregnated with the coupling agent 3-MPTMS and treated with AgNP s1 and s2 revealed better resistance to microbial attachment and biofilm formation (Figure 11). Specifically, the treated limestone substrate, coded with L in the graphic and silanized with 3-MPTMS and AgNPs (Figure 11), displayed a notable decrease in biofilm formation, which decreased approximately by 4 logs as compared to the limestone control or the limestone sample treated solely with 3-MPTMS (Figure 11). Similarly, the treatment applied to the travertine substrate involving 3-MPTMS and AgNP s2 led to decreased biofilm formation ability, surpassing both the travertine control and the travertine sample treated only with 3-MPTMS. The comparative analysis depicted in the graphic (Figure 11) illustrated that both travertine and limestone samples solely treated with 3-MPTMS exhibited limited antibiofilm ability against B. subtilis, comparable to the untreated control stone specimens. The reason is attributed to the protective role of the coupling agent in preventing water absorption. The presence of siloxanes on the stone substrates leads to the formation of a Si–O–Si network on the treated surface [55]. More precisely, for both the untreated travertine or limestone samples, as well as the stone samples treated with 3-MPTMS, no discernable differences in biofilm formation were observed.

It is noteworthy that the higher porosity of the travertine substrate evident on comparison with limestone (Figure 8) facilities greater penetration, going deeper inside the substrate, and thus, silver nanoparticles should also be deposited deeply, but these nanoparticles do not count on the antimicrobial/antibiofilm activity, and this is why this activity is lower. These observations were reinforced by SEM images, which revealed higher roughness and greater pores in travertine compared with limestone (Figure 8c).

**Figure 12:**
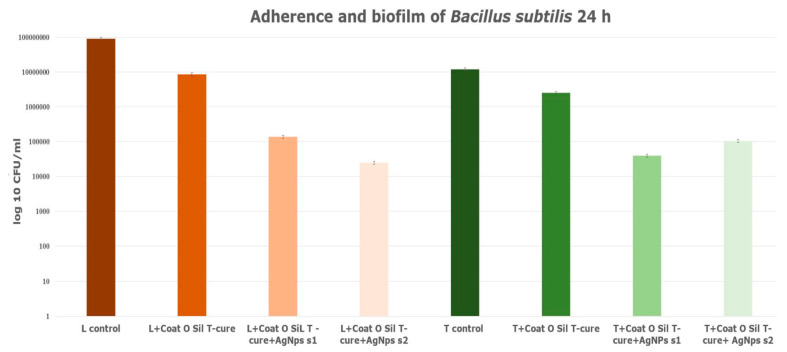
Adherence biofilm of *B. subtilis* on limestone and travertine treated with CoatOSil T-Cure.

The limestone specimens treated with CoatOSil T-Cure as a silanization agent exhibited a substantial biofilm inhibitory ability of approximately 3 logs as compared to the untreated control (Figure 12).

The presence of AgNPs on the coated surface increased the anti-biofilm activity of the CoatOSil-T-Cure-coated stone samples (Figure 12). Moreover, the presence of AgNP s1 yielded higher resistance in treated travertine potentially influenced by the specific type and dimensions of silver nanoparticles. A comparison of values obtained from the treatment of stones with 3-MPTMS (Figure 11) and those from treatment with CoatOSil T-Cure (Figure 12) on stone substrates indicated that the latter exerts a comparatively less influence. It is plausible that 3-MPTMS is a more suitable candidate for porous stone. The discrepancy suggests that the coupling agent may not establish optimal adherence on a stone substrate when compared to 3-MPTMS and that nanoparticles may not fully interact with all thiol groups provided by the coupling agent.

**Figure 13:**
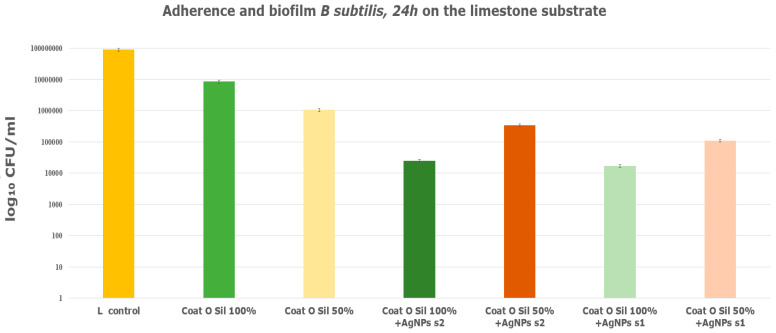
Adherence of *B. subtilis* biofilm on limestone treated with various concentrations of CoatOSil T-Cure and various AgNPs.

The graphic (Figure 13) exhibits the adherence and biofilm formation of *B. subtilis* on limestone treated with solely 100% CoatOSil T-Cure, respectively, with the mixing of 50% CoatOSil T-Cure and 50% isopropyl alcohol (IPA) driven to different results. When comparing the results of samples treated with 100% CoatOSil T-Cure coupling agent and AgNPs, for both concentrations, with the limestone control, we noted an increase in the inhibition of *B. subtilis* biofilm in terms of CFU/mL values of around 3–3.8 logs (Figure 13). Likewise, when comparing the same samples of limestone treated solely with 100% CoatOSil T-Cure, there was a rise in CFU/mL values, indicating an increasing inhibition of *B. subtilis* biofilm with 2.8 to 3 values on a logarithmic scale (Figure 13). Therefore, it is evident that for samples treated with 100% CoatOSil T coupling agent, AgNPs play a significant role in enhancing resistance against *B. subtilis*.

However, the same observation was not apparent for samples treated with a mixture of coupling agent and isopropyl alcohol (IPA). Both samples treated with AgNP s1 and s2 exhibited an increase in resistance against *B. subtilis* compared to the control sample, with CFU/mL values approximatively 2.5 and 3 times higher on a logarithmic scale (Figure 13). Nevertheless, when comparing the obtained values for the AgNP-treated samples with those for the limestone sample treated solely with the coupling agent and IPA mixture, the decrease in resistance in terms of CFU/mL was only 0.5 to 1 units on a logarithmic scale. This suggests that, in this case, the presence of isopropyl alcohol may act as a biocide, but it does not enhance the action of AgNPs. The alcohol could help to penetrate the stone substrate, including the coupling agent, but this means that AgNPs will be absorbed deeper into the stone structure and will lead to an overall lower AgNP concentration on the surface and thus lower microbiological activity.

**Figure 14:**
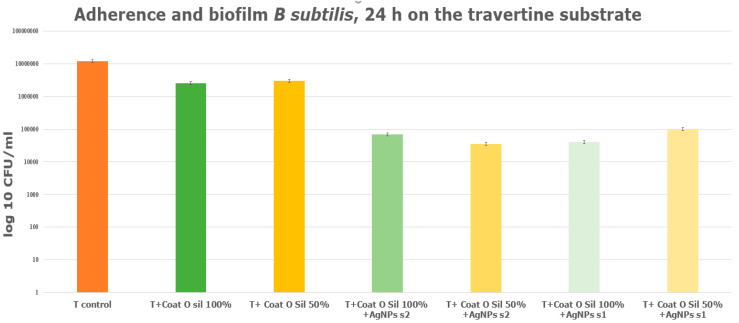
Adherence of *B. subtilis* biofilm on travertine treated with various concentrationd of CoatOSil T-Cure.

The investigation of travertine samples subjected to different concentrations of CoatOSil T-Cure treatment revealed, as shown in the graphic (Figure 14), that the presence of alcohol does not exert a discernable influence on the resistance of *B. subtilis.* This observation holds true when comparing travertine samples treated with 100% CoatOSil T-Cure with those treated with 50% CoatOSil T-cure + 50% isopropyl alcohol. Furthermore, in the travertine samples treated with 100% CoatOSil T-Cure and AgNPs, various concentrations exhibited decreasing resistance to biofilm formation, with CFU/mL values in the range of 2 to 3 on a logarithmic scale. Additionally, travertine samples treated with 100% and 50% Coat OSil T-Cure exhibited similar antimicrobial activity, suggesting that the presence of alcohol does not significantly affect porous stone characterized by open pores. Probably, the coupling agent is adsorbed inside the structure of stones, and less quantity remains on the substrate to link with AgNPs. These insights are grounded in the comparable values obtained for travertine (Figure 13) treated with various concentrations of the coupling agent. It is noteworthy that the antimicrobial activity of travertine treated with AgNPs displayed marginal discrepancies among the samples, with CFU/mL values being approximately 1 or 2 times on a logarithmic scale compared to travertine treated only with 100% CoatOSil T-Cure (Figure 14).

**Figure 15:**
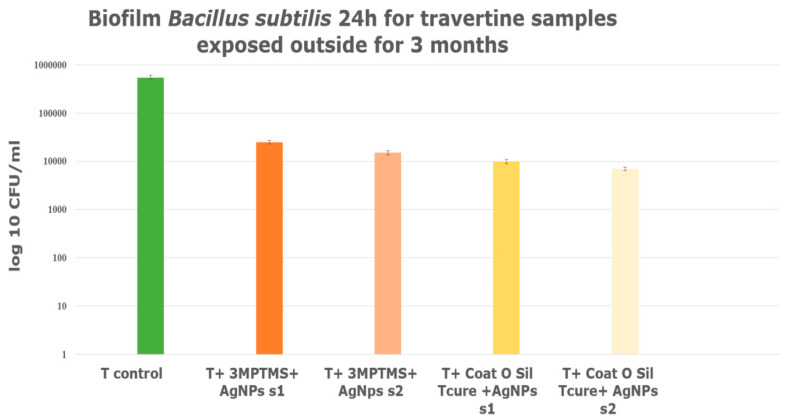
Biofilm formed during exposure outside, for 3 months; travertine treated with different coupling agents and AgNPs.

A series of natural stones samples of travertine treated with AgNPs and exposed outdoors, following the same protocol as in the laboratory, was treated with coupling agents and silver nanoparticles. Subsequently, they were exposed to the outside to all conditions: sunrays, rains, and wind. All treated samples exhibited antimicrobial resistance, implying that the treatments have a beneficial effect. It is evident that samples treated with AgNP s2 demonstrated markedly enhanced antimicrobial resistance in comparison to samples treated with AgNP s1 (Figure 15). The travertine samples treated with both coupling agents, 3-MPTMS and CoatOSil T-Cure, in conjunction with AgNPs, exhibited distinct responses. Notably, the sample treated with CoatOSil T-Cure and AgNP s2 revealed a slightly improved resistance against *B. subtilis*, with a CFU/mL about 2.8 times on a logarithmic scale in comparison to the control sample (Figure 15). It is important to highlight that samples treated with 3-MPTMS also displayed comparable values, approximately around 2 or 2.5 on a logarithmic scale. Both coupling agents can be recommended to be used for porous stones; in this case, the coupling agent with stronger affinity is CoatOSil T-Cure.

## 5. Conclusions

Stone modification using silanization agents is widely used, but mostly, it is just for consolidation and surface impermeabilization. The solution proposed in this paper is devoted to inducing additional antimicrobial and antibiofilm properties. It is important to mention that mercapto groups have a high affinity for gold and silver surfaces, and this is important because these nanoparticles will be strongly attached on these modified surfaces.

Our results prove that the substrate and the used silanization agent have an important impact on the final antimicrobial/antibiofilm activity. When using 3-MPTMS as a silanization agent and AgNPs, the obtained coating showed the best antimicrobial properties when applied to limestone and travertine specimens. The treated limestone samples showed better inhibition potential against *B. subtilis* attachment and biofilm development as compared to travertine specimens treated with the same coating mixture.

The silanization agent CoatOSil T-Cure showed similar effects on the antimicrobial properties of coated stones, regardless of the two types of AgNPs used. Based on the comparative analyses conducted on the two types of porous stone with various coupling agents, it was observed that the correct selection of the coupling agent is crucial depending on the substrate characteristics. Moreover, the results achievable through AgNP fixation can be significantly improved. These findings were concluded upon the results obtained after exposure of the treated stones outside. By rigorously selecting a coupling agent based on its physico-chemical features, an appropriate quantity of nanoparticles (NPs) can be used, ensuring long-lasting efficiency and preventing wastage of nanoparticles and consequently not affecting the environment. Further works have to be conducted one for long-time exposure of treated specimens in environmental conditions to elucidate for how long the antibiofilm effect is maintained and for what microbial species range.

## Figures and Tables

**Table 1:** Stone characteristics.

Stones Used for Treatment, Classification by Type of Rocks				
Stone Sample No.	Type of Stone	Rock Group	Main Component(s)	Main Characteristics
1	Travertine	Sedimentary	Calcium carbonate	High porosity, open and closed pores, water absorption by weight > 10%
2	Limestone	Sedimentary	Calcium carbonate (aragonite), silica, fossil components	Medium porosity, water absorption by weight < 10%

**Table 2:** Type of AgNP solutions used in the tests and their characterization.

Sample No.	Code of AgNPs	Type of Synthesis Method	Concentration of AgNPs	Properties of AgNPs
S1	AgNPs RT	Classical reduction method at room temperature	10 ppm	Small sizes and different shapes of nanoparticles, mainly truncated
S2	AgNPs HT	Solvothermal method at 260 °C	1000 ppm	Spherical shapes and large sizes of nanoparticles

**Table 3:** Type of coupling agent used for treating stone.

Siloxanes—Coupling Agents	Formula
3 mercapto propyl trimethoxysilane (3 MPTMS)	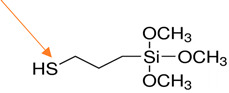
OAT O SIL-T CURE	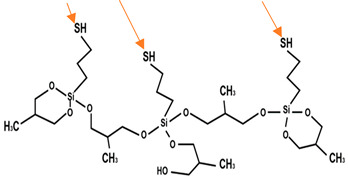

## Data Availability

Data are contained within the article.
